# Medical students’ preclinical service-learning experience and its effects on empathy in clinical training

**DOI:** 10.1186/s12909-021-02739-z

**Published:** 2021-05-26

**Authors:** Yi-Sheng Yang, Pei-Chin Liu, Yung Kai Lin, Chia-Der Lin, Der-Yuan Chen, Blossom Yen-Ju Lin

**Affiliations:** 1 Department of Environmental and Occupational Medicine, National Taiwan University Hospital, Taipei, Taiwan, Republic of China; 2 Department of Anesthesiology, Taipei Veterans General Hospital, Taipei, Taiwan, Republic of China; 3 Department of Surgery, Chiayi Branch, Taichung Veterans General Hospital, Chiayi, Taiwan, Republic of China; 4 Division of Cardiovascular Surgery, Taichung Veterans General Hospital, Taichung, Taiwan, Republic of China; 5 Department of Otolaryngology-Head & Neck Surgery, China Medical University Hospital, Taichung, Taiwan, Republic of China; 6 School of Medicine, China Medical University, Taichung, Taiwan, Republic of China; 7 Rheumatology and Immunology Center, China Medical University Hospital, Taichung, Taiwan, Republic of China; 8 College of Medicine, China Medical University, Taichung, Taiwan, Republic of China; 9 Department of Medical Humanities and Social Sciences, School of Medicine, College of Medicine, Chang Gung University, No.259, Wenhua 1st Rd., Guishan Dist., Taoyuan City, 33302 Taiwan, Republic of China

**Keywords:** Service learning, Curriculum-based service team, Extracurricular service team, Empathy, Clinical training, Clerkships

## Abstract

**Background:**

Service learning (SL) is an educational methodology presumed to help medical students be more empathetic and compassionate. We longitudinally investigated the level of empathy in medical students and how preclinical SL experience was related to their level of empathy in their clinical clerkships.

**Methods:**

Our cohort comprised fifth-year medical students engaged in clerkships as part of a 7-year medical programme at one medical school in Taiwan. Surveys were conducted at the beginning of the clerkship in September 2015 (T1) to collect data on the medical students’ preclinical SL experience in curriculum-based service teams (CBSTs) and extracurricular service teams (ECSTs) and their SL self-efficacy, demographic characteristics, and empathy level. Subsequently, three follow-up surveys were conducted once every 3 months to determine the empathy level of the students during their clinical clerkships (T2–T4). Seventy students who returned the written informed consent and completed the baseline (T1) and two or more follow-up surveys (T2–T4) were included in our analysis with the response rate of 34%. In total, 247 responses across the 1-year clerkship were analysed. Descriptive statistics, paired *t* tests, and generalised estimating equations were employed.

**Results:**

Our study revealed that changes in empathy level in the dimensions of *perspective taking*, *compassionate care*, and *standing in patients’ shoes* in their clinical clerkships. Relative to that at T1, their empathy decreased in *perspective taking* and *compassionate care* at T2–T4 but increased in *standing in patients’ shoes* at T3. Additionally, our study verified the positive effect of medical students’ preclinical SL experience in CBSTs and ECSTs on empathy in terms of *compassionate care* and *perspective taking*, respectively, but not on that of *standing in patients’ shoes*.

**Conclusions:**

Separate investigations into subconstructs of empathy, such as *perspective taking*, *compassionate care*, and *standing in patients’ shoes*, in medical students may be necessary for exploring the various driving forces or barriers to developing empathy in medical students. Moreover, SL experience through both CBSTs and ECSTs at medical academies may have positive effects on medical students’ empathy in their clinical clerkships and should be promoted at medical schools.

## Background

In medicine, empathy is defined as a practitioner’s ability to perceive and understand a patient’s emotional state and clearly articulate the patient’s feelings [1]. The Association for Medical Education in Europe recommended that empathy be included as a key criterion in the evaluation of academic performance and professionalism [2]. Studies have indicated a correlation between empathy and good clinical performance [3], specifically in communicative ability [4], low burnout rates [5], and improved well-being [6]. Moreover, empathy has been associated with high patient satisfaction [7–13], high medical compliance [11, 14–17], and favourable treatment results [8, 10, 14, 18–20] and reduced medical costs [21], medical errors [22], and medical malpractice [23–26]. Because of the critical role of empathy in medical training, the cultivation of empathy in medical students has been a recent topic of interest. Medical educators have endeavoured to propose methods for improving empathy in medical students; for example, by improving students’ communication skills, incorporating perspectives of medical humanities, and reforming medical curricula [1, 27]. A study in Singapore reported that community service and socialisation may be key factors affecting the demonstration of empathy in medical students [28].

Service learning (SL) is a pedagogical process that enriches conventional coursework through the inclusion of activities outside the classroom that meet the needs of the community [29]. Implementing SL early in medical training as a form of community-based learning may provide a formative experience that influences the perspectives of medical students as they enter clinical clerkships [30]. SL can be traced back to 1938, when John Dewey first argued that education in prosocial values should not occur in the abstract, such as in classrooms or lectures; rather, students should learn from real-world experience [31, 32]. SL incorporates domains of education, such as knowledge, attitudes, skills, intentions, and relationships through a process-oriented participatory approach [33]. SL facilitates the transformation of student knowledge and attitudes in the context of holistic care through work with community organisations [34] to help students become more socially responsible, patient-oriented practitioners [35]. SL is also considered a method for nurturing the service commitments of medical students while promoting interactions with the communities they serve [36, 37]. SL has been argued to transform student perspectives on humanism in medicine, including the perspectives of students who respect and empathise with the struggles and strengths of their patients and peers [38]. For example, the Loyola University Chicago Stritch School of Medicine proposed a 5-year Global Health Fieldwork Fellowship track that was grounded in clinical and service learning, doing so to foster the development of prosocial values, such as professionalism, advocacy, and social justice in their medical students [39]. SL programmes involving work with the homeless have also enhanced the professional and personal education of medical students by improving their understanding of biopsychosocial problems and by developing their empathy, compassion, and social awareness [40, 41].

The effectiveness of SL is often evaluated by assessing students’ qualitative reflections immediately following such programmes. However, in such evaluations, common-method biases may give rise to instructors posing leading questions; thus, instructors may either implicitly or explicitly make assumptions regarding participants’ responses. Few studies have longitudinally explored the effects of SL experience on empathy in medical students. Therefore, we quantitatively and longitudinally examined how preclinical SL experience at medical schools was related to medical students’ empathy in clinical training. Two methods of analysis were employed. First, we longitudinally investigated the change in the level of empathy of medical students during their clinical clerkships. Second, we explored the relationship of medical students’ preclinical SL experience with their empathy during their clerkships.

## Methods

This study administered a prospective web-based survey to a cohort of medical students at one medical school in Taiwan.

### Study participants

Our study involved a group of fifth-year medical students participating in clinical clerkships as part of a 7-year medical programme at a medical school in Taiwan starting in September 2015. After recruitment at the end of August 2015, 140 of 206 medical students (68%) who agreed to participate in the study returned written informed consent forms.

### Measurement

#### Medical students’ preclinical SL experience

In higher education, SL can be fostered using various formats and is often classified as an academic course, either credited or noncredited. A curriculum-based service team (CBST) refers to a team that participates in community service activities included in credited academic courses, whether a required or elective course [37, 42, 43], with explicit learning objectives [44]. Students in CBSTs perform various activities in small groups in structured learning environments that incorporate preparation, action, reflection, and celebration under the supervision of course instructors [45]. Depending on the instructors’ curriculum design, CBSTs perform several community services that focus on one or several themes during one or more academic semesters. By contrast, extracurricular service teams (ECSTs) are composed of students in voluntary and interdisciplinary societies who perform community services that are outside the curriculum and for which academic credit is not given. ECSTs are typically led by senior students and advised by faculty members; this format is also common in Western medical schools [46, 47]. For medical students, the service themes of CBSTs and ECSTs include caring for older people, people with disabilities, or inpatients; junior student tutoring; health education and promotion; or offering free clinical services in urban, rural, or overseas regions.

In this study, medical students’ preclinical SL experience was measured in terms of their membership of CBSTs and ECSTs and their service intensity in these teams during the preclinical stage of medical school (years 1 to 4 in a 7-year programme). One questionnaire item recorded whether the medical students were members of a CBST or ECST. In terms of service intensity, respondents reported the number of service themes they were engaged in and the average number of hours they spent on planning and preparation, action, reflection, and celebration per service theme for both CBSTs and ECSTs. In another questionnaire item, the students’ reported their perceived SL self-efficacy at the preclinical stage of medical school by using a 5-point scale, with a higher score indicating greater SL efficacy.

#### Empathy of medical students in clinical clerkships

Several instruments have been developed to measure empathy. The Jefferson Scale of Empathy was applied in this study because it was designed not for the general population but rather for patient care situations [48]. Empathy ratings for medical students were measured using the Jefferson Scale of Physician Empathy-Student version (JSPE-S), which comprises 20 items rated on a 7-point Likert scale, ranging from 1 (*strongly disagree*) to 7 (*strongly agree*) [3, 48]. The JSPE-S was translated into traditional Chinese and evaluated by English language specialists and Chinese language specialists from Taiwan. An exploratory factor analysis using the principal component method (Kaiser–Meyer–Olkin statistic = 0.919) was performed first because of potential differences in responses due to perceived cultural differences (the questionnaire was originally developed in a Western country). Two items were omitted because their factor loadings were lower than 0.5 after an exploratory factor analysis using varimax rotation with Kaiser normalisation (item 1: physicians’ understanding of their patients’ feelings and the feelings of their patients’ families does not influence medical or surgical treatment; item 18: physicians should not allow themselves to be influenced by strong personal bonds between their patients and their family members). Three common factors were identified, namely *perspective taking* (nine items), *compassionate care* (seven items), and *standing in patients’ shoes* (two items), and their Cronbach’s α values were 0.922, 0.935, and 0.844, respectively. These three factors were adopted from Hojat et al. [3]; *perspective taking* refers to the students’ perception of patients’ perspectives, *compassionate care* refers to the students’ understanding of patients’ affective states during inpatient care, and *standing in patients’ shoes* refers to the students’ ability to understand patients’ experiences. Notably, these items’ locations in the three factors in this study were similar to the item distributions verified in Japanese [49] and Korean [50] studies of medical students and a US study of students of osteopathic medicine [51].

Detailed information is provided in Table 1. For further analysis, three factor scores were calculated for the three factors by using regression methods.

**Table 1:** Factor structures for perceived empathy of medical students in clinical clerkships (*N* = 247)

Items	Question	Mean	SD	Factor loadings		
				Perspective taking	Compassionate care	Standing in patients’ shoes
Item 16	Physicians’ understanding of the emotional status of their patients, as well as that of their families, is one important component of the physician–patient relationship.	5.563	0.960	0.860		
Item 17	Physicians should try to think like their patients in order to render better care.	5.417	1.020	0.837		
Item 15	Empathy is a therapeutic skill without which the physician’s success is limited.	5.190	1.217	0.810		
Item 20	I believe that empathy is an important therapeutic factor in the medical treatment.	5.510	1.100	0.792		
Item 13	Physicians should try to understand what is going on in their patients’ minds by paying attention to their nonverbal cues and body language.	5.405	1.103	0.754		
Item 2	Patients feel better when their physicians understand their feelings.	5.749	0.934	0.709		
Item 10	Patients value a physician’s understanding of their feelings which is therapeutic in its own right.	5.130	1.243	0.699		
Item 4	Understanding body language is as important as verbal communication in physician–patient relationships.	5.729	0.977	0.672		
Item 5	A physician’s sense of humor contributes to a better clinical outcome	5.389	1.014	0.671		
Item 14	I believe that emotion has no place in the treatment of medical illness. (R)	5.619	1.266		0.859	
Item 8	Attentiveness to patients’ personal experiences does not influence treatment outcomes. (R)	5.360	1.327		0.857	
Item 12	Asking patients about what is happening in their personal lives is not helpful in understanding their physical complaints. (R)	5.514	1.331		0.852	
Item 9	Physicians should not try to stand in their patients’ shoes when providing care to them. (R)	5.555	1.357		0.848	
Item 7	Attention to patients’ emotions is not important in history taking. (R)	5.445	1.384		0.838	
Item 11	Patients’ illnesses can be cured only by medical or surgical treatment; therefore, physicians’ emotional ties with their patients do not have a significant influence in medical or surgical treatment. (R)	5.150	1.364		0.788	
Item 19	I do not enjoy reading nonmedical literature or the arts. (R)	5.304	1.335		0.701	
Item 3	It is difficult for a physician to view things from patients’ perspectives (R)	3.879	1.260			0.901
Item 6	Because people are different, it is difficult to see things from patients’ perspectives. (R)	3.510	1.300			0.899

Note: (1) The item # in Table 1 is marked the same as that in the original version of the Jefferson Scale of Physician Empathy-Student version (JSPE-S) for readability and comparison across studies. (2) Each item was measured on a 7-point Likert scale ranging from 1 (*strongly disagree*) to 7 (*strongly agree*). (3) (R) refers to reversed scores for the item (ie, 1 → 7, 2 → 6, 3 → 5, 4 → 4, 5 → 3, 6 → 2, and 7 → 1)

#### Background information

The medical students’ personal background information, namely sex and age, were recorded.

### Data collection

Surveys were conducted at the beginning of the clerkship in September 2015 (T1), followed by three follow-up surveys in December 2015 (T2), April 2016 (T3), and August 2016 (T4). Data on the following were gathered: preclinical SL experience, specifically —membership of and service intensity in both CBST and ECST; demographic characteristics, specifically sex and age; and baseline empathy level (T1). Follow-up surveys were conducted to determine the empathy level of the medical students during their clinical clerkships (T2–T4). The completion of the follow-up surveys was voluntary, and those who completed the baseline (T1) and two or more follow-up surveys (T2–T4) were included in this study. In total, 70 medical students (response rate 34% = 100% × 70/206) with 247 responses were included in our analysis.

### Statistical analysis

Descriptive statistics of the medical students’ demographic characteristics, preclinical SL experience, and longitudinal empathy level during their clerkships were analysed. Paired *t* tests were performed to analyse the dynamics of the medical students in terms of empathy level across the three dimensions of *perspective taking*, *compassionate care*, and *standing in patients’ shoes*; comparisons were performed for T1–T2, T1–T3, T1–T4, T2–T3, T2–T4, and T3–T4.

Generalised estimating equations (GEEs) were used to analyse the repeated measures of empathy in medical students according to selected predictors. Regression of the dependent variable for selected independent variables was conducted using a GEE method for the following reasons [52, 53]. First, a repeated measures analysis was employed for the dependent variable. Second, several missing values were identified for the predictors. Third, robust standard estimates were available for performing consistent and accurate tests of statistical significance. Fourth, the application of the Quasi-likelihood Information Criterion (QIC) reflected the relative efficacy of the proposed model in fitting the data. In this study, three GEEs were used for each dependent variable; a repeated measures analysis was performed on medical students’ empathy factor scores for *perspective taking*, *compassionate care*, and *standing in patients’ shoes*, and the students’ demographic characteristics and preclinical SL experience were applied as predictors. Notably, variables with skewness and kurtosis values greater than 2 and 7 [54], respectively, were transformed (e.g. the students’ age and total hours serving in CBSTs and ECSTs). Statistical analyses were performed using SPSS 25.0 (IBM Corporation, Chicago, IL, USA).

## Results

In total, 70 medical students (men: 36, 51%; women: 34, 49%) with response rate 34% were included in our study, with an average age of 23 years. Among them, 54 students (77%) served as CBST members, engaging in an average of six service themes and spending 15 h per theme. In total, 42 students (60%) served as ECST members, engaging in an average of five service themes and spending 31 h per theme. The students reported an average SL self-efficacy score of 3.4 on a 5-point scale. Detailed information is presented in Table 2.

**Table 2:** Medical students’ personal backgrounds and preclinical SL experience (*N* = 70)

Variables	Scale	Mean (Frequency)	SD (Percentage)
**Demographic characteristics**			
Sex	Male	(36)	(51)
	Female	(34)	(49)
Age		23.486	2.005
**Preclinical SL experience**			
CBST membership	No	(16)	(23)
	Yes	(54)	(77)
CBST service intensity: the number of service themes the student engaged in		6.069	6.250
CBST service intensity: the average time spent per service theme (hrs)		14.987	30.177
ECST membership	No	(28)	(40)
	Yes	(42)	(60)
ECST service intensity: the number of service themes the student engaged in		4.579	10.485
ECST service intensity: the average time spent per service theme (hrs)		31.029	66.420
Perceived SL self-efficacy (5-point scale)		3.400	0.824

Note: SL: service learning; CBST: curriculum-based service team; ECST: extracurricular service team

Three dimensions of empathy level (*perspective taking*, *compassionate care*, and *standing in patients’ shoes*) were measured over four periods (T1, T2, T3, and T4). The T1, T2, T3, and T4 evaluations were completed by 70, 64, 65, and 48 students, respectively (Table 3). Paired *t* tests were performed for individual comparisons at two time points; the results revealed that the scores for *perspective taking* at T2, T3, and T4 were lower than those at T1, and the scores for *compassionate care* at T2, T3, and T4 were significantly lower than those at T1 (*p* < 0.05). By contrast, the score for *standing in patients’ shoes* at T1 was significantly lower than that at T3 (*p* < 0.05). Detailed information is listed in Table 3.

**Table 3:**
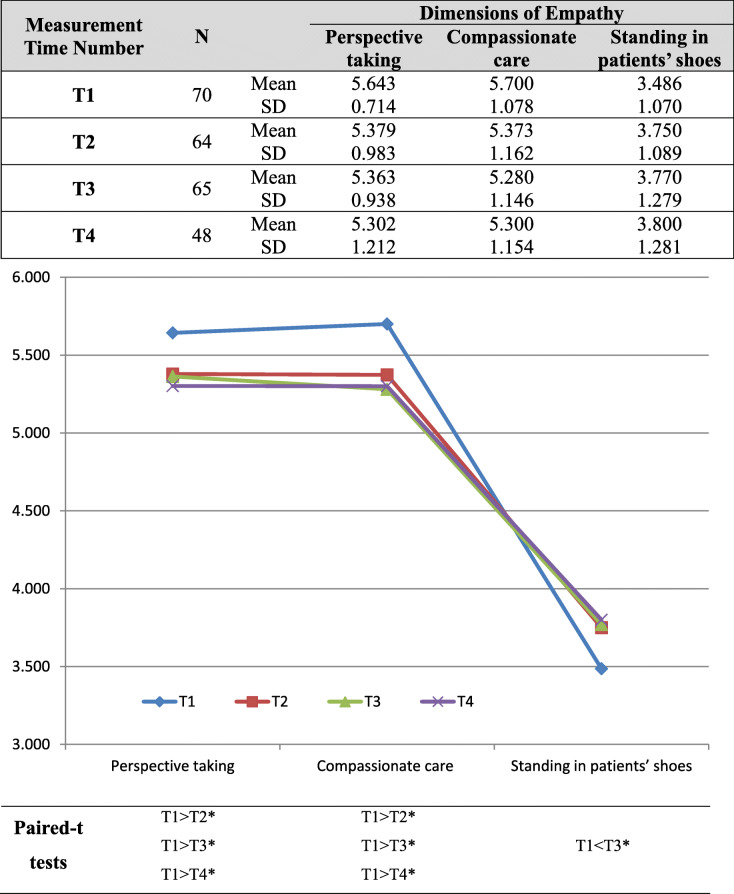
Medical students’ empathy level across four timelines (N = 247)

Note: *p < 0.05

Table 4 illustrates the GEE results for the effects of predictor variables in repeated measures of medical students’ empathy level in the individual dimensions of *perspective taking*, *compassionate care*, and *standing in patients’ shoes*. The results revealed that (1) the students’ preclinical experience measured by total hours serving in ECSTs was positively related to their empathy in terms of *perspective taking* (*p* < 0.05) and (2) that in CBSTs was positively related to students’ empathy in terms of *compassionate care* (*p* < 0.05). However, the medical students’ preclinical SL experience in CBSTs and ECSTs was not related to their empathy in terms of *standing in patients’ shoes* (*p* > 0.05).

**Table 4:** Relationships of medical students’ preclinical SL experience with their empathy level in clinical clerkships: Generalised estimating equation modelling

Variables	Dimensions of Empathy								
	Perspective taking			Compassionate care			Standing in patients’ shoes		
	Estimates	SE	Sig	Estimates	SE	Sig	Estimates	SE	Sig
Intercept	−1.074	1.253	0.391	−1.880	1.176	0.110	1.375	1.215	0.258
**Preclinical SL experience**									
CBST membership (default = no)	−0.054	0.272	0.843	−0.132	0.309	0.669	−0.065	0.248	0.792
CBST service intensity: total hours served^a^	−0.019	0.013	0.161	0.031	0.012	0.012	−0.011	0.016	0.482
ECST membership (default = no)	0.041	0.247	0.868	0.492	0.253	0.052	0.162	0.233	0.487
ECST service intensity: total hours served^b^	0.008	0.003	0.013	0.003	0.003	0.274	0.006	0.004	0.101
Perceived SL self-efficacy	0.247	0.137	0.071	−0.186	0.120	0.121	0.194	0.150	0.197
**Demographic characteristics**									
SEX (default = male)	−0.095	0.194	0.625	−0.070	0.200	0.728	−0.191	0.181	0.290
AGE^c^	−0.329	1.067	0.758	−1.826	1.098	0.096	1.737	1.082	0.109
Model fit	QIC = 1020.826 QICC = 252.786			QIC = 846.987 QICC = 246.639			QIC = 1123.132 QICC = 245.870		

Note:

1. SL: service learning; CBST: curriculum-based service team; ECST: extracurricular service team

2. ^a^CBST service intensity: total hours served was calculated by multiplying the number of service themes a student was engaged in by the average number of hours spent per CBST service theme. The original value of total hours engaged in CBST was transformed using the square root value to meet the assumption of normality

3. ^b^ECST service intensity: total hours served was calculated by multiplying the number of service themes a student engaged in by the average number of hours spent per ECST service theme. The original value of total hours engaged in ECST was transformed using square root value to meet the assumption of normality

4. ^c^The original AGE value was transformed using LN(LN(LG(X))) because of the violation of the normality assumption

## Discussion

After longitudinally tracking the empathy levels of 70 medical students during their clerkships for 1 year (i.e. four iterations:T1–T4), our study revealed various changes in empathy level across the dimensions of *perspective taking*, *compassionate care*, and *standing in patients’ shoes*. Specifically, relative to that in T1, the students’ empathy decreased in *perspective taking* and *compassionate care* at T2–T4 but increased in *standing in patients’ shoes* at T3. In addition, our study verified the positive relationship of medical students’ preclinical SL experience in CBSTs and ECSTs on *compassionate care* and *perspective taking*, respectively, but not on *standing in patients’ shoes* in terms of empathy level during their clinical clerkships.

Previous studies have employed a cross-sectional approach and the JSPE-S to determine the empathy level of medical students during medical school, and diverse findings have been reported; for example, in terms of changes in medical student empathy during medical school, several studies have reported decreasing levels [55–60], several have reported increasing levels [49, 50, 61, 62], and others have reported no statistically significant differences [63]. Notably, the cross-sectional designs applied in these studies could not account for the developmental trajectory of medical students or allow for repeated testing. To address these limitations, Costa et al. [64] conducted longitudinal research to collect data on the empathy of preclinical medical students; their results revealed no decline in the medical students’ empathy over time. However, we argue that summed empathy scores might hamper the identification of individual items or distinctive characteristics across the many dimensions of empathy. Therefore, by employing longitudinal tracking of the subconstructs of empathy in medical students, our study discovered that empathy decreased in the dimensions of *perspective taking* and *compassionate care* but increased in that of *standing in patients’ shoes* (Table 3, paired *t* tests, *p* < 0.05). A previous study employed the JSPE at the beginning (pretest) and end (posttest) of medical students’ third year of clerkship at a medical school in the United States, noting statistically significant declines in empathy scores for items 2, 10, 11, 12, and 15, which related most closely to the dimensions of *perspective taking* and *compassionate care* [65]; these results are consistent with the current findings (Tables 1 and 3). We content that separate investigations into the subconstructs of empathy, such as *perspective taking*, *compassionate care*, and *standing in patients’ shoes*, in medical students enable future investigations of various potential driving factors of or barriers to the development of empathy with respect to these subconstructs.

Most importantly, our study verified the benefits of CBST in cultivating empathy with respect to *compassionate care* during clinical clerkships (*p* < .01). The items under *compassionate care* indicate how much respondents regard health and illness from a holistic perspective; that is, the medical practitioner considers medicine from not only a biomedical perspective (as a science) but also from a psychosocial perspective (as an art) [48]. CBSTs are typically incorporated into various educational domains by instructors to influence students’ planning and preparation, behaviour, reflection and demonstration, and assessment and celebration [32]. By discussing learning objectives with instructors and community partners in academic courses, students can enhance their awareness and gain deeper insights into social problems to identify social needs and acquire strong problem-solving skills [66–68]. Therefore, we argue that the explicit learning objectives of CBSTs are related to cognitive ability, as reflected by academic performance, civic engagement, social skills, and improved attitudes towards the self [69–71]. Therefore, helping medical students regard patient services from a systematic perspective and holistically understand patient concerns may benefit medical students and help them perform compassionate care during their clinical training.

Furthermore, we revealed that engagement in ECSTs helps medical students cultivate empathy with respect to *perspective taking* during their clinical clerkship (*p* < .05). The items related to the subconstruct of *perspective taking* emphasised empathy in interpersonal relationships when determining patients’ cognitive status [48]. ECSTs are typically led by senior students and advised by school faculty [46] and are typically composed of students from many disciplines who collaborate to solve community problems. Such teamwork may provide opportunities for students to build relationships with their partners, faculty, the community, or people they serve to reinforce school and community ties and encourage collaboration [67, 72, 73]. Moreover, an interprofessional service and learning experience was reported to generate the greatest effect on medical students’ perceptions of their competency and training in other disciplines [74]. Moreover, students can apply problem-solving and learning cycles to promote deep learning through service delivery [75]. Therefore, we maintain that ECSTs may provide opportunities to improve medical students’ social awareness and cultural competency in relation to their student peers from other disciplines and their community partners from a diversity of backgrounds [47], thereby increasing their empathy in *perspective taking* towards patients following their clinical clerkships.

Although our study revealed that medical students’ empathy in terms of *standing in patients’ shoes* increased in clinical clerkships at T3 (Table 3), we could not verify the potential benefits of SL experience on medical students’ empathy in terms of standing *in patients’ shoes* (Table 4). Santiago et al. [76] compared medical students’ empathy at two medical schools and argued that student empathy could be increased by implementing earlier and more intensive contact with patients under the supervision of skilled tutors. We contend that clinical training is essential for medical students to learn how to manage real-life situations, and through clinical training, medical students are encouraged to regularly interact with real patients to enhance their practical knowledge [77]. By learning to understand patients’ experiences, medical students gradually develop the ability to provide exceptional medical care during clinical training.

This study has several limitations. First, the service intensity (i.e. the number of service themes engaged in and the average time spent per service theme) of medical students’ SL experience was aggregated across various services themes in CBSTs and ECSTs because of the limited sample size. Thus, we were unable to analyse the effectiveness of individual service themes in SL experience. Second, the SL experience of medical students was analysed only in the context of the preclinical stage of their medical school, and their self-reported information on SL experience may have been affected by recall bias. Third, the successful implementation of CBSTs and ECSTs requires careful planning, the deliberate articulation of rationales and expectations, the strengthening of community relationships, and the conduct of iterated reviews and revisions to the programme [78]. Insufficient time, resources, and effort for effective implementation may burden students and educators with record keeping, transportation, and scheduling [79]. Thus, we could not confirm the quality of the CBST and ECST values reported by the medical students in our study, which may have confounded the effectiveness of SL experience. Fourth, the inclusion of medical students from only one medical school and the small sample size (response rate 34%) with potential respondent bias may hamper the generalizability of the study findings. We got the medical students’ written informed consents around 68% of the cohort of medical students in the study recruitment stage. However, in order to catch the dynamics of the medical students’ empathy levels in their clinical training given the participants’ voluntary survey responses, so as to enhance our study contributions on exploring the SL effects on empathy longitudinally, we might make the trade-off to include the small size (response rate 34%) in our data analysis. Moreover, to assess how medical students’ SL effectiveness is sustained over time, we might call for action to routinely collect or record the medical students’ well-being and soft skills such as empathy into their learning portfolio, especially at their socialization in the clinical training, even after clerkships or postgraduate medical training.

## Conclusion

Our study quantitatively and longitudinally explored how medical students’ preclinical SL experience was related to their empathy in clinical clerkships. We found that medical students’ empathy level in their clinical clerkships exhibited different patterns (increasing or decreasing) across the dimensions of *perspective taking*, *compassionate care*, and *standing in patients’ shoes*. Moreover, our study revealed that SL experience in both CBSTs and ECSTs at medical academies may have positive effects on medical students’ empathy in their clinical clerkships and should be promoted at medical schools.

## Data Availability

The data included in this study, with departmental information and student identities removed, are available for use on reapproval by any eligible research ethics committee.
